# A genomic inference of the White Plymouth Rock genealogy

**DOI:** 10.3382/ps/pez411

**Published:** 2019-07-15

**Authors:** Y Guo, M Lillie, Y Zan, J Beranger, A Martin, C F Honaker, P B Siegel, Ö Carlborg

**Affiliations:** 1 Department of Medical Biochemistry and Microbiology, Uppsala University, Uppsala 75123, Sweden; 2 Department of Animal and Poultry Sciences, Virginia Tech, Blacksburg, VA 24061; 3 The Livestock Conservancy, Pittsboro, NC 27312

**Keywords:** domestication, ancestry, admixture, phenotype–genotype interface, chickens

## Abstract

Crossing of populations has been, and still is, a central component in domestication and breed and variety formation. It is a way for breeders to utilize heterosis and to introduce new genetic variation into existing plant and livestock populations. During the mid-19th century, several chicken breeds that had been introduced to America from Europe and Asia became the founders for those formed in the USA. Historical records about the genealogy of these populations are often unclear and inconsistent. Here, we used genomics in an attempt to describe the ancestry of the White Plymouth Rock (WPR) chicken. In total, 150 chickens from the WPR and 8 other stocks that historical records suggested contributed to its formation were whole-genome re-sequenced. The admixture analyses of the autosomal and sex chromosomes showed that the WPR was likely founded as a cross between a paternal lineage that was primarily Dominique, and a maternal lineage where Black Java and Cochin contributed in essentially equal proportions. These results were consistent and provided quantification with the historical records that they were the main contributors to the WPR. The genomic analyses also revealed genome-wide contributions (<10% each) by Brahma, Langshan, and Black Minorca. When viewed on an individual chromosomal basis, contributions varied considerably among stocks.

## INTRODUCTION

The domestication of the chicken from the jungle fowl has resulted in a wide variety of populations across the world (FAO, 2015) with purposes ranging from ceremonial rites, cock fighting, and fancy plumage to production of meat and eggs for human consumption (Smith and Daniel, 1975). That differences in breeding schemes have contributed to considerable genetic diversity across domesticated populations (Wong et al., 2004) is hardly surprising, given their phenotypic diversity. Less genetic diversity in current commercial chicken populations than across the ancestral breeds is likely due to the limited number of incorporated breeds (Muir et al., 2008). However, much of the standing genetic variation contributed by the founder breeds remains in populations that have been highly selected for many generations for meat or egg production, or both (Sheng et al., 2015; Lillie et al., 2018). A possible explanation for this diversity is that these stocks were developed via admixtures of diverse populations prior to the initiation of the breeding programs for meat or egg production. Traces of admixtures are sometimes obvious in, for example, plumage and egg color; however, genome-wide traces of such admixtures have been unexplored. The advent of genomics-based strategies has allowed for more detailed information about the admixtures, which is of both basic and applied interest. Such information has the potential to guide genomics-based crossbreeding schemes and across-population breeding-value predictions. The historical records about the genealogy of chicken stocks are often anecdotal, incomplete, or totally missing. Genomics provides a tool to explore the admixture histories and to evaluate the validity of available historical records.

The Plymouth Rock is a chicken in which the barred variety was originally developed in the USA in the mid-19th century. The precise origin, timeline, and stocks involved in its development are not clear. To our knowledge, the first mentioning of Plymouth Rocks is from an exhibition at the 1849 American Poultry Show (Robinson, 1913). They later reappeared at a show in 1869 (Corbin, 1879) before becoming formally accepted into the American Poultry Association (**APA**) *Standard of Excellence* in 1874 (APA, 1947). The White Plymouth Rock (**WPR**) is a plumage color variety that was subsequently developed from the barred variety and recognized as a breed by the APA in 1888 (APA, 1947). Documentation about the genealogy of the WPR provides somewhat conflicting information regarding which breeds contributed to its formation (Corbin, 1879; Dohner, 2001). For example, Plymouth Rock chickens presented at the 1869 show resulted from a cross between a Dominique cock and either a Black Cochin or a Black Java hen (Procter, 1911; Scrivener, 2014). The base may have been broader due to the known intermingling of chickens from several breeds, including White Cochin, Dark and White Brahma, Black Java, Langshan, Dorking, Black Minorca, White-faced Black Spanish, and Dominique (Procter, 1911; Scrivener, 2014). Corbin (1879), in his discussion of the distinction between breeding Plymouth Rocks for utility and show, recognized inbreeding depression such as back crossing and sib matings in what he termed “in-and-in breeding.” Regardless, the Plymouth Rock became a popular farmed chicken and the WPR, assumed to be one of the main sources for the commercial broiler industry (Gordy, 1974), was also one of the sources for commercial brown egg production (Fulton et al., 2016).

The objective of this study was to use genomics to explore the admixture history of the 8 stocks generally assumed the sources of the WPR. This, in turn, allowed evaluation of the validity of available historical records of the WPR, dating back to the APA in 1888.

## MATERIALS AND METHODS

The 8 stocks evaluated as contributors to the WPR were Black Cochin, Buff Cochin, Partridge Cochin, Black Minorca, Black Java, Langshan, Light Brahma, and Dominique. The WPR was represented by the high (**HWS**) and low (**LWS**) selected Virginia BW lines. These lines were founded in 1957 as the progeny of crosses between 7 partially inbred lines of WPR (Siegel, 1962; Dunnington et al., 2013). Since then, they have been closed populations subjected to bidirectional selection for high or low BW at 8 wk of age. Pedigree analysis showed that 29 (of 44) and 30 (of 51) of the 1957 founders for the HWS and LWS lines, respectively, still contributed to generation 48 (Márquez et al., 2010). This is also reflected in the high levels of genomic diversity that have been maintained both within and across the lines (Sheng et al., 2015; Lillie et al., 2018) despite the single trait selection regime. Therefore, the Virginia BW lines were considered as representative for the WPR breed, as of the mid-20th century in the USA. Our thesis was that because selection was for the quantitative trait with moderate heritability, their admixtures would be similar and thus serve as replicates for the admixture analyses. In total, 150 chickens were used. They included generation 40 of the Virginia BW lines (HWS n = 29 and LWS n = 30). The donor stocks, Black Cochin (n = 10), Partridge Cochin (n = 4), Buff Cochin (n = 9), Dominique (n = 10), Black Java (n = 10), Langshan (n = 13), Black Minorca (n = 14), and Light Brahma (n = 21), were obtained from populations at poultry exhibit shows, suppliers, and small farm flocks in the United States. Table 1 presents phenotypic information of these stocks. All procedures were carried out in accordance with the guidelines established by Virginia Tech Institutional Animal Care and Use Committee.

**Table 1.** Phenotypes of stocks used in genomic analyses.

	Phenotype			
			Shank	
Stock	Feathering	Comb	Feathered	Color
White Plymouth Rock	Early	Single	Clean	Yellow
Black Cochin	Late	Single	Feathered	Mixed^2^
Buff Cochin	Late	Single	Feathered	Mixed^2^
Partridge Cochin	Late	Single	Feathered	Mixed^2^
Black Minorca	Early	Single	Clean	Black
Light Brahma	Late	Rose	Feathered	Yellow
Black Java^1^	-	Single	Clean	Mixed^2^
Dominique^1^	-	Rose	Clean	Yellow
Langshan^1^	-	Single	Clean	Black

1 From American Poultry Association (1947).

2 Epidermal-dermal.

### Genotyping

Libraries for sequencing the chickens from the Virginia lines were prepared using the Illumina TrueSeq protocol and sequences (on average 34.3 × genome coverage) obtained by paired-end sequencing (2 × 150 bp) on an Illumina HiSeq X at the SciLifeLab SNP&SEQ Technology platform (Uppsala, Sweden). Libraries for sequencing the samples from the other 8 stocks were prepared using an optimized version of a *Tn5*-based protocol (Picelli et al. 2014) for low-cost, high-throughput preparation of individual sequencing libraries (∼1€/library). The genomic DNA was fragmentized and tagged using *Tn5* transpose purified from a plasmid available from AddGene (http://www.addgene.org/.pTXB1-Tn5; ID60240) (Picelli et al. 2014). Dual indexes were attached during PCR amplification and subsequent size selection was performed using AMPure XP beads (Beckman: A63881). The libraries were sequenced to, on average, 4.3 × coverage on an IIlumina Hiseq X Ten sequencer at ANOROAD (Beijing, China). All samples in this study were individually sequenced. Table 2 and Supplemental Figure S1 provide information on sequencing depth for each population. Obtained sequence reads were mapped against the ICGSC Gallus_gallus-5.0 reference genome (Nov. 2011) using BWA (Li and Durbin, 2010). SNP calling was performed using GATK (v3.7) (McKenna et al., 2010) using the best practice pipeline. Small indels and variants with more than 2 alleles were removed. Quality control was implemented using VCFtools (Danecek et al., 2011) to filter out reads that did not meet the following criteria: low mapping quality variants (genotypes called < 0.5, minor allele count < 3, minimum quality score < 20). The sequencing data generated for this study are available in the NCBI Short Read Archive (https://www.ncbi.nlm.nih.gov/sra), accession number: PRJNA552722.

**Table 2.** Population information.

	Average sequencing depth	Females	Males	Samples
**Founder stocks**				
Buff Cochin	3,8	1	8	9
Black Cochin	4,0	0	10	10
Partridge Cochin	5,2	0	4	4
Light Brahma	3,8	3	18	21
Black Minorca	4,3	0	14	14
Black Java	4,1	5	5	10
Langshan	4,5	10	3	13
Dominique	4,7	8	2	10
	**Total**	**27**	**64**	**91**
**WPR stocks**				
HWS	34,0	19	10	29
LWS	34,6	21	9	30
	**Total**	**40**	**19**	**59**

Information about the evaluated stocks (total number of samples, number of females and males, and average sequencing depth). Founder stocks: stocks evaluated as potential founders for the White Plymouth Rock (WPR); HWS and LWS: the Virginia high and low BW selected lines.

### Ancestry Haplotype Painting

Ancestry haplotype painting was performed using ChromoPainter (Lawson et al., 2012). It used haplotype similarity information of the individuals of the analyzed populations to infer a “coancestry matrix” revealing ancestral relationships among the analyzed individuals. The sharing of ancestors among populations resulted in extended shared segments of DNA where each chromosome could ultimately be broken down into a series of such ancestral haplotypes. For each individual and segment, the donor source was assigned a specific color (or paint) according to its origin. The average copy proportion of each ancestral stock per locus was calculated separately for HWS and LWS, and the mean values were used to paint the chromosome.

### Admixture History and Percentage Analyses

Admixture events were inferred using an approach based on genome-wide patterns of ancestry to infer which source groups were likely involved and the fine-scale information about the resulting mixtures across the genomes. The method implemented in the software Globetrotter (Hellenthal et al., 2014) relies on genetic data alone and does not require a priori specification of surrogates for the original sources of the target. Haplotype output from ChromoPainter was used as input to estimate whether the target population was likely to descend from admixture events of the ancestral “surrogate” stocks. Here, the 8 non-WPR stocks, suggested by historical records as possible contributors to the WPR, were used as “surrogates,” whereas the HWS and LWS were used as separate targets for whole-genome and chromosomal-separate analyses. Mean copy proportions for each “surrogate” stocks were calculated by averaging the proportion of admixture source times mixing coefficient.

### Sex Chromosome Analyses

First, the sex of individuals from the 8 non-WPR stocks was determined from the individual whole genome sequence data. Only females are expected to have reads mapping to the W chromosome because they are the heterogametic sex (ZW), whereas the males are the homogametic sex (ZZ), in chicken. For each individual, the missing rate on the W chromosome was obtained using VCFtools (Danecek et al., 2011) and if <40%, the chicken was scored as female. To evaluate the accuracy of the procedure, the 59 individuals from Virginia lines with known sex were tested, and all were classified correctly. In total, 27 females and 64 males were identified across the 8 stocks (Table 2).

Next, after the sex assignment, heterozygous sites on the W chromosome were marked as missing and a secondary filtering was applied to only keep individuals with at least 80% call rate. The ancestry analysis was performed for using the ChromoPainter (Lawson et al., 2012)/Globetrotter (Hellenthal et al., 2014) pipeline, as for the autosomal chromosomes. Only females were used for the W, and males for the Z, chromosome analyses.

## RESULTS

### Whole-Genome Autosomal Chromosome Admixture Analyses

**Figure 1.**
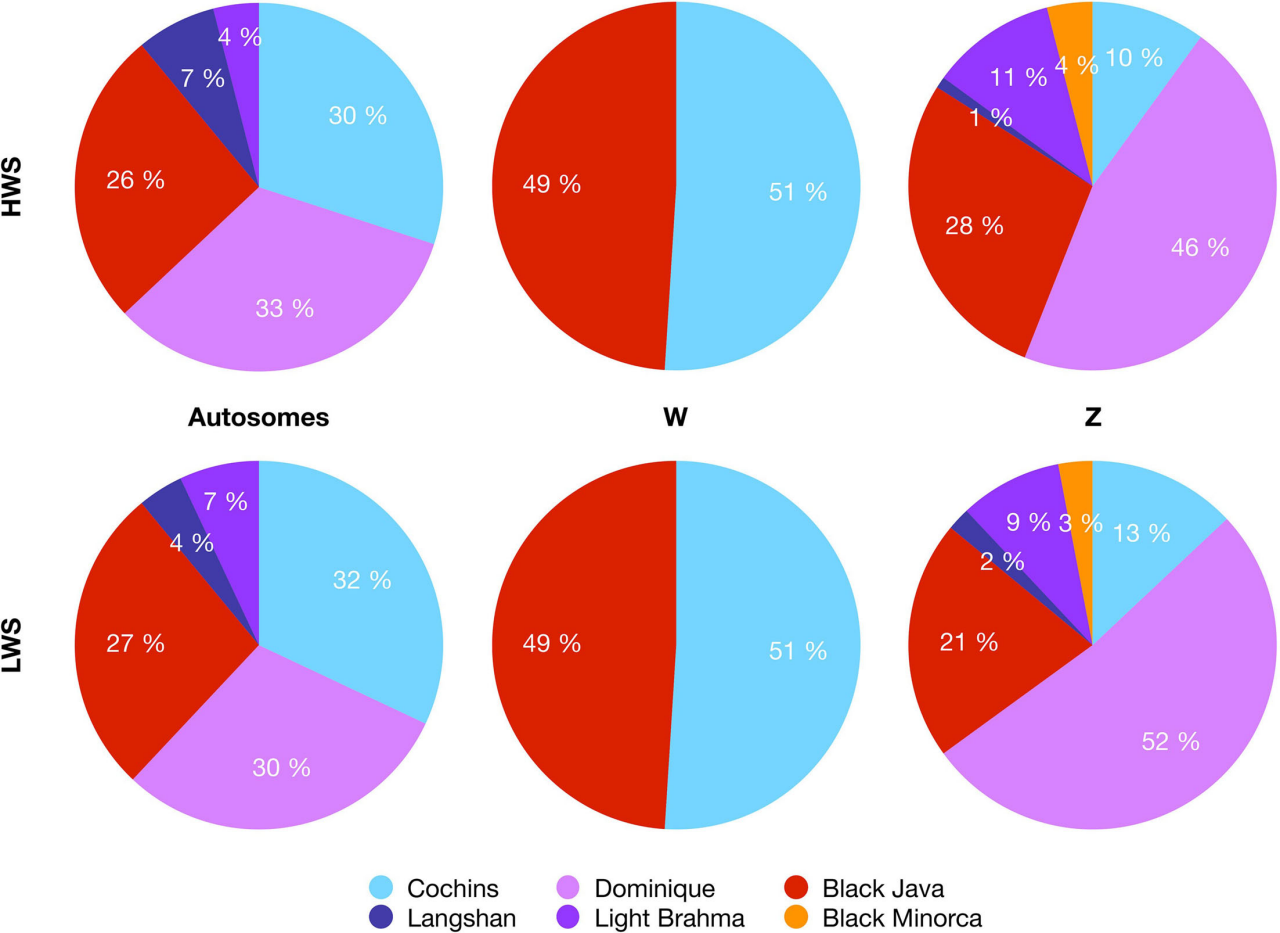
The admixture proportions on autosomes and W and Z chromosomes of the founder stocks in the White Plymouth Rock (WPR) Virginia high (HWS) and low (LWS) BW selected lines.

**Figure 2.**
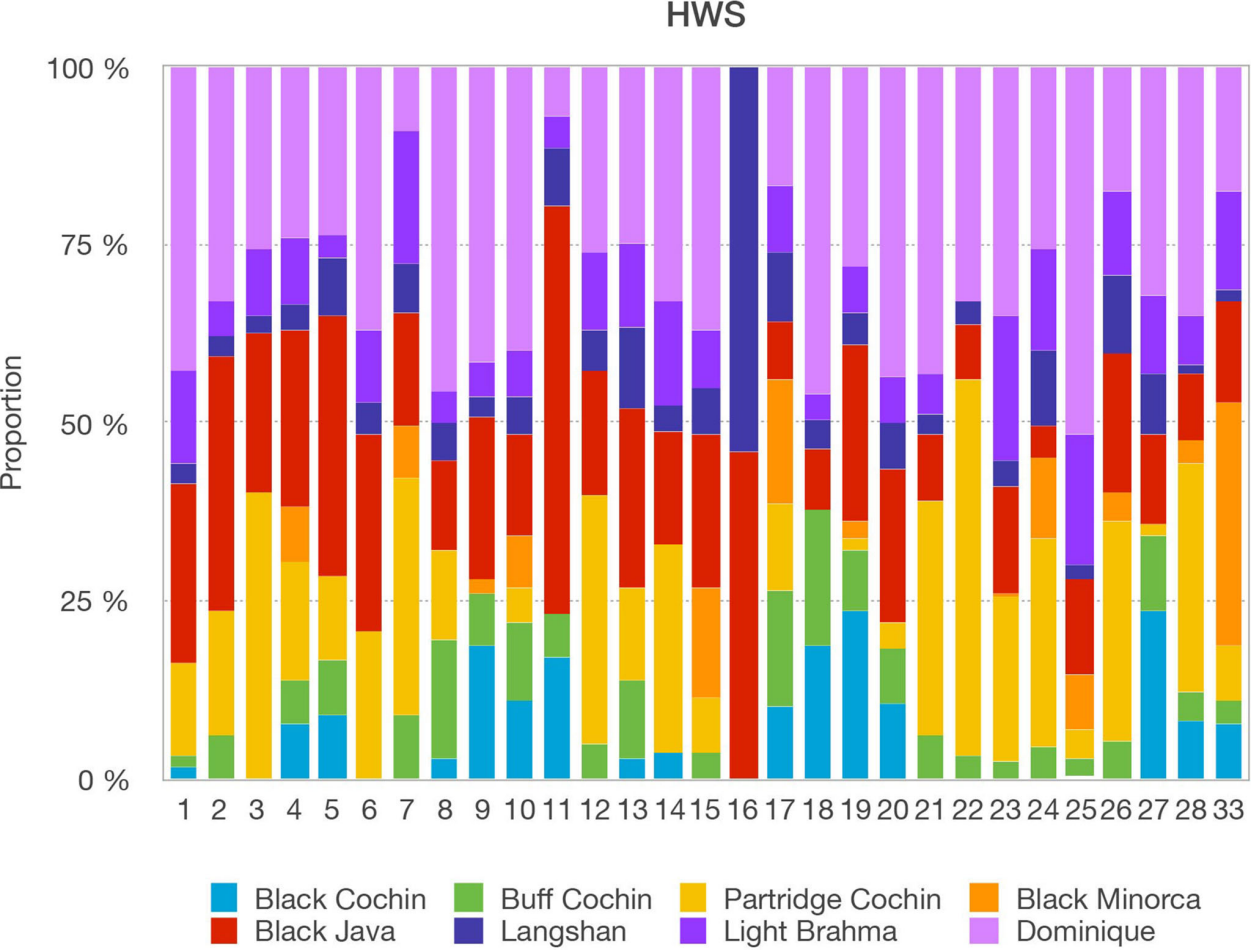
Chromosome paintings illustrating the variation in stock contributions to the autosomal genome of the Virginia high BW selected line (HWS). The colors represent the respective recipient copied from different ancestry sources (purple = Light Brahma, green = Buff Cochin, dark blue = Langshan, light blue = Black Cochin, pink = Dominique, orange = Black Minorca, red = Black Java, gold = Partridge Cochin). Chromosomes are displayed along the x-axis from chromosome 1 to chromosomes 28 and 33. Copy proportions are displayed on the y-axis.

**Figure 3.**
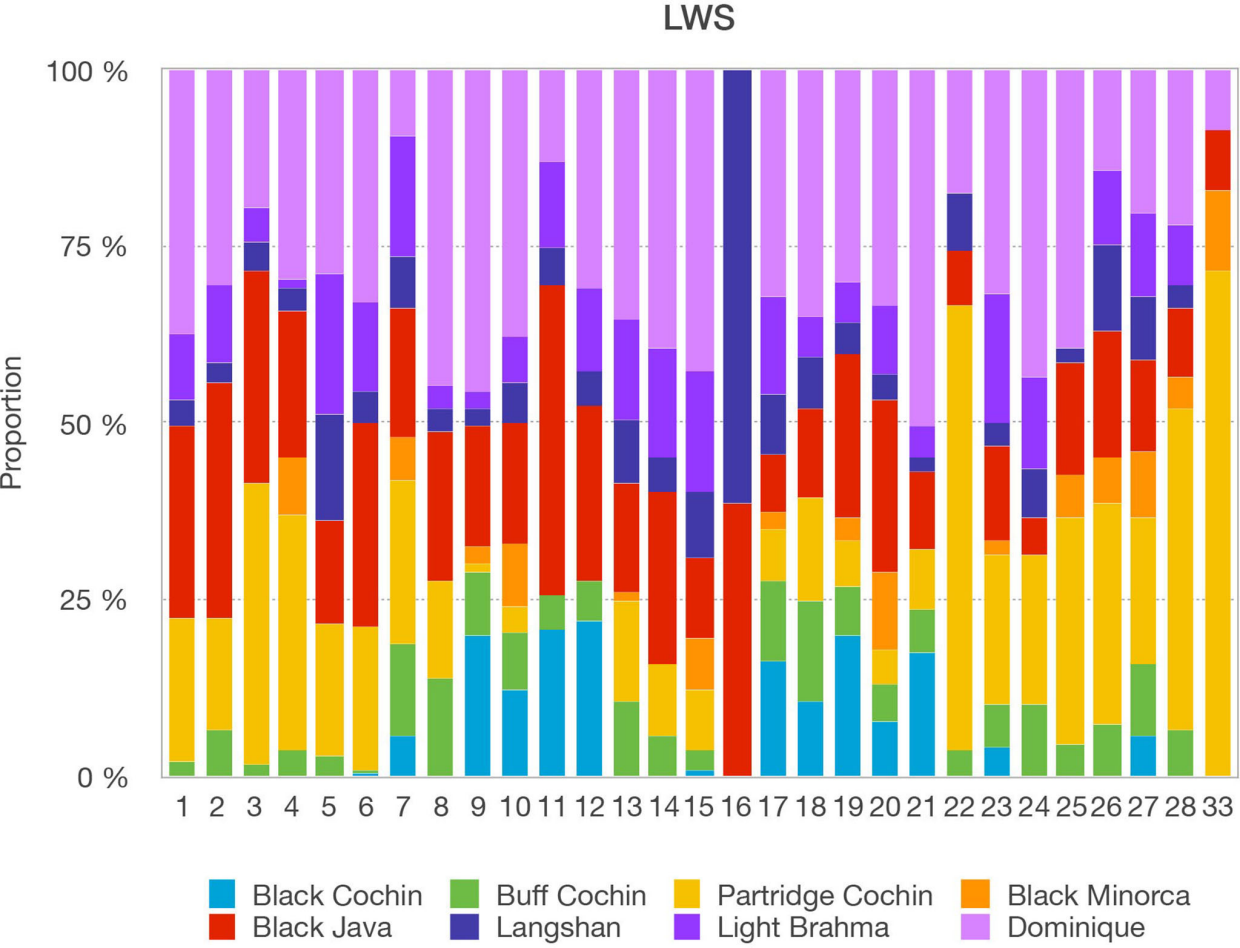
Chromosome painting showing the component difference among chromosomes for the Virginia low BW selected line (LWS). The colors represent the respective recipient copied from different ancestry sources (purple = Light Brahma, green = Buff Cochin, dark blue = Langshan, light blue = Black Cochin, pink = Dominique, orange = Black Minorca, red = Black Java, gold = Partridge Cochin). Chromosomes are displayed along the x-axis from chromosome 1 to chromosomes 28 and 33. Copy proportions are displayed on the y-axis.

Admixture analyses for the HWS and LWS lines (Figure 1) revealed only minor differences between them. This shows that the divergent selection for high and low body weights in these lines had little impact on the estimation of the genealogy of the WPR. Although the genome analyses detected admixture, they could not provide a clear inference of the date and “best-guess” sources of either single or multiway admixtures. Using ChromoPainter data, the proportions of genome-wide contributions of the donor stocks to HWS and LWS were calculated separately (Figures 2 and 3). The 4 major donors to the Virginia BW lines, together contributing 89% of the autosomal genome, were Dominique, 2 of the Cochins (Buff & Partridge), and Black Java. The respective values for HWS were 33, 30, and 26%. For LWS, they were 30, 32, and 27%. In addition to these 3 major donor groups, other stocks contributing more than 0.1% on the genome-wide scale were Light Brahma (4% HWS, 7% LWS) and Langshan (7% HWS and 4% LWS).

### Individual Autosomal Chromosome Admixture Analyses

Separate admixture analyses were performed for 29 autosomal chromosomes including chromosome 1 through chromosomes 28 and 33 (Figures 2 and 3). The estimation of breed proportions failed for chromosomes 30 (222 kb), 31 (169 kb), and 32 (252 kb) due to their small sizes, and they were therefore omitted from the results. Overall, the pattern resembled that of the genome-wide analysis, with large contributions by Dominique, Black Java, and the Cochins to most chromosomes. However, both the proportions and donor sources varied among the chromosomes (Figures 2 and 3). Variation was also observed between the HWS (Figure 2) and LWS lines (Figure 3), suggesting that they, at least in part, resulted from the strong divergent selection for BW applied to the Virginia lines.

Contributions by 4 or more stocks were present on most autosomal chromosomes, the exception being chromosome 16, where only the Langshan and Black Java contributed in almost equal proportions. Although Black Minorca and Black Cochin made small contributions on the genome-wide scale, the chromosome-specific analyses revealed larger contributions (up to 25%) by these stocks on one or several chromosomes.

### W-Chromosome Admixture Analysis

Historical records suggest uneven contributions of the stocks to the maternal and paternal lineages of the WPR. The W-chromosome was therefore analyzed to reveal the maternal lineage of the admixtures. Only the Black Java (49%) and Cochins (51%) contributed to this chromosome (Figure 1). This finding is consistent with these stocks contributing only to the maternal lineage, as the overall contributions to the autosomal genome are close to half of that (Figure 1).

### Z-Chromosome Admixture Analysis

To explore the ancestry in the paternal lineage of the WPR, an admixture analysis was also performed for the Z-chromosome using only male data. The analysis revealed that Dominique contributed about 50% of the Z chromosome (46/52% HWS/LWS), with contributions also from Black Java (28/21% HWS/LWS), Cochins (10/13% HWS/LWS), Light Brahma (11/9% HWS/LWS), Black Minorca (4/3% HWS/LWS), and Langshan (1/2% HWS/LWS) (Figure 1). As these results only reflect a single chromosome, the exact breed proportions will reflect admixtures as well as the effects of selection and other population genetic forces acting in the WPR lines. However, the larger contributions by Dominique, Light Brahma, and Black Minorca to the Z-chromosome than to the autosomal genome—together with no contributions to the W-chromosome—strongly suggest that these stocks only contributed to the paternal lineage. That the Cochins contributed less to the Z-chromosome than the autosomal genome and the W-chromosome strongly suggests that these breeds contributed only to the maternal lineage. The contribution by Black Java to the Z-chromosome was marginally smaller than to the autosomal genome, making it higher than expected given that the autosomal and W-chromosome analyses together imply that it only contributed to the maternal lineage. The Langshan made a smaller contribution to the Z-chromosome than expected based on the autosomal and W-chromosome analyses; however, because its overall contribution is so small, the estimates are likely to be too imprecise to conclude more than it is expected to contribute to the paternal lineage.

## DISCUSSION

Over the last century, poultry breeding has made progress in developing elite populations by utilizing genetic variation from domestic stocks across the world. The WPR has been one of the major contributors to the modern broiler and brown egg layer due to its rapid growth, hardiness, and good reproduction, compared to other chicken breeds (Gordy, 1974; Fulton et al., 2016). According to historical records, it was developed in the USA in the mid-19th century as a cross between multiple stocks that had earlier been introduced to America. However, which stocks were crossed and how and at which proportions was not known with certainty. Here, whole-genome re-sequencing and admixture analyses were used to evaluate the proposed historical scenarios regarding the origin of the WPR. Our inference is limited to a sample of breeds including those with the strongest support from historical records, and its consistency with these suggests that only minor contributions from other sources may have been missed. Although the depth of sequencing varied between the samples and stocks, we do not consider it to influence the broad overall inferences of breed contributions to the WPR. Strict standards were used for SNP filtering, and breed contributions were based on haplotype analyses where possible genotyping errors in individual SNPs were likely less influential.

### Divergence in Ancestry Between HWS and LWS

Only minor differences were observed in ancestry between the HWS and LWS on the genomic scale. This suggests that the strong divergent selection for 56-D BW in the Virginia lines between 1957 and 1997 did not have any major overall impact on the admixture signals relative to the ancestral WPR population. The differences were more pronounced on individual chromosomes, where the ancestry differed markedly at many locations along the genome. Such differences are likely the result of population genetic processes, such as selection for high and low BW, as well as drift. Subsequent analyses of the Virginia BW lines may provide more insights to the relationship between the ancestry in the specific regions and the BW differences between the lines that have been the result of the long-term selection experiment and historical recombination. Generating the haplotype mosaic in these regions is also likely to be informative for future fine mapping efforts with these lines.

### Dominique is the Major Contributor to the WPR

The admixture analyses showed that Dominique was the major contributing stock to the WPR, which is consistent with the consensus of the available historical records (APA, 1947; Dohner, 2001). The Dominique was an important contributor across autosomes of the HWS and LWS lines, the exceptions being chromosomes 7 and 11, plus 16 to which it did not contribute. The W-chromosome analyses suggest that the contribution of the Dominique to the WPR is entirely on the paternal side, which is consistent with the available historical records (Dohner, 2001).

### The Maternal Lineage of the WPR is Dominated by Black Java and Cochin

The admixture analyses of the W-chromosome showed that only 2 stocks, the Black Java and Buff Cochin, contributed to this chromosome at near equal proportions, 49 and 51%, respectively. This is consistent with these stocks contributing approximately 1/4 each to the autosomal genome.

Cochins, originating in China, are large chickens with feathered shanks. They were first used for exhibition purposes in Europe due to their excessive plumage, rather than because they were good layers and meat producers. They had an important role in the development of female parent lines of broilers (Gyles, 1989). The Black Java is also a heavy breed that was originally used for both egg and meat production, and historical records suggest that it made important contributions to other breeds in the Americas such as the Jersey Giant and Rhode Island Red (Ekarius, 2007). The Black Java, together with Langshan, made an interesting contribution to chromosome 16, where the majority of genes have a demonstrated role in immune responses, including the major histocompatibility complex (Miller and Taylor, 2016). These 2 breeds may have important contributions to the immune gene repertoire of WPR, but further, in-depth investigations would be required to understand this fully.

### Contributions by Langshan, Light Brahma, and Black Minorca to the WPR

The whole genome and Z-chromosome admixture analyses (Figure 1) illustrate that 3 additional stocks, Langshan, Light Brahma, and Black Minorca, also contributed to the WPR through the paternal lineage. The parsimonious explanation is that the initial crossings to generate the Barred Plymouth Rock were made using males that were genetically and phenotypically primarily Dominique. The genomic analyses suggest that these males were not purebred Dominique, but rather from a population into which smaller proportions of the other stocks had been previously introgressed. This explanation is consistent with historical records suggesting that such mixtures were common in the USA during the mid-19th century (Corbin, 1879; Procter, 1911). An alternative explanation suggested by Procter (1911) is the near-simultaneous development of 2 or more lineages, with introgression on the male side prior to line crossing in the final formation of the breed.

### General Comments

The formation of the WPR (APA, 1947; Dohner, 2001) occurred prior to the rediscovery of Mendelism. Although introgression was prevalent, as was experimentation to develop new breeds, poultry breeding during this period emphasized purity of blood lines. Selection was based on phenotype, and breed standards were established by the APA beginning in 1873. Chickens having undesirable features were disqualified as potential breeders. By these means, desirable alleles and allelic combinations were enriched in breeding flocks. The WPR, one of the foundation breeds for the commercial broiler (Gordy, 1974), came from white chicks that periodically hatched from matings of standardbred Barred Plymouth Rocks. This sport occurred in flocks that had supposedly introgressed White Birmingham, which were recessive white (*cc*), into their population and then repeatedly backcrossed to eliminate progeny with white feathers (Hawes, 1988). Periodically, white chicks would appear from the barred population. Being recessive, they would “breed true,” and a white variety of the Barred Plymouth Rock was easily produced.

It appears that with our current knowledge of Mendelian genetics and the relative molecular contributions of the 8 stocks referred to as sources of the WPR, an outline of the development of the Plymouth Rock was quite linear. Recessive white (*cc*) and sex-linked early feathering (*k-*) are recessives to the dominant allele (Hutt, 1949), as is yellow skin (W*Y) (Eriksson et al., 2008). Single comb (*rrpp*) is also a recessive with complimentary gene action between pea comb located on chromosome 1 (Sato et al., 2010) and rose comb located on chromosome 7 (Imsland et al., 2012). In addition, rose comb is associated with reduced male fertility issues (Imsland et al., 2012), and single comb males benefited from preferential matings due to their higher positions in the social hierarchy (Guhl and Ortman, 1953; Siegel and Dudley, 1963). Producing a chicken with a clean shank could be addressed over several generations. This was because feathered shank (ptilopody), located on chromosome 13, was multi-allelic (Somes, 1992), and there were modifiers that had to be addressed (Hutt, 1949). Early writers confirm that selection of clean-legged birds was necessary during the foundation of the Barred Plymouth Rock (Procter, 1911). Even more complex was shank color because of epidermal and dermal issues, including penetrance, multiple alleles, and modifiers (Dorshorst et al., 2010).

The molecular analyses presented here reflect the influence of introgressions on the overall genome and specific chromosomes, using the WPR as the model. Although analyses reflected a major contribution of the Dominique, the proportion of its contributions varied among chromosomes. This was particularly evident for the minor contribution to chromosome 7, on which rose comb is located, and no contribution to chromosome 16.

### Conclusions

Our genomic analyses show that the major contributors to the WPR breed were Dominique males and Black Java and Cochin females. Smaller contributions by Langshan, Light Brahma, and Black Minorca were also found. The current WPR breed likely originates from a stock where Dominique, Langshan, Light Brahma, and Black Minorca were intermixed prior to crossings involving equal proportions of females from Black Java and Cochin lineages. Overall, this finding is consistent with available historical records. In addition, it resolves the proportions by which the suggested stocks contributed to the WPR, as well as which of them contributed to the paternal and maternal lineages of the breed.

## Supplementary Material

pez411_Supplemental_Figure
