# Supplementary figures and images for: A genomic inference of the White Plymouth Rock genealogy
Source: Poult Sci. 2019 Jul 15;98(11):5272–80. doi: 10.3382/ps/pez411 (PMC6863967; doi:10.3382/ps/pez411)

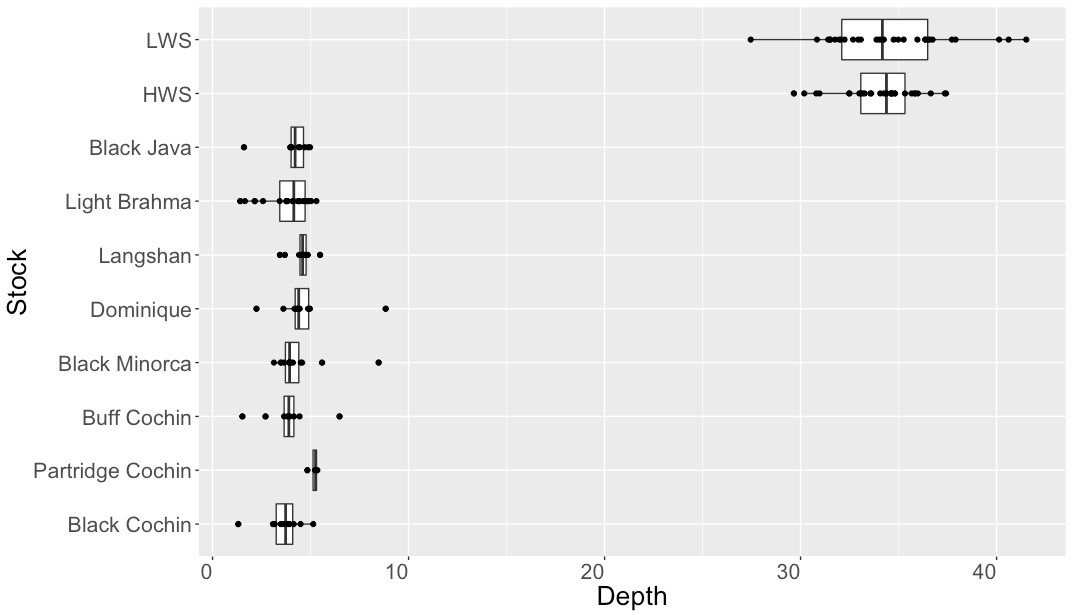

Supplement: pez411_Supplemental_Figure [file pez411_supplemental_figure.jpeg]
